# Supplementary material for: Delayed gut microbiota maturation in the first year of life is a hallmark of pediatric allergic disease
Source: Nat Commun. 2023 Aug 29;14:4785. doi: 10.1038/s41467-023-40336-4 (PMC10465508; doi:10.1038/s41467-023-40336-4)
Supplement: Supplementary file 1 — Supplementary Information [file 41467_2023_40336_MOESM1_ESM.pdf]

| Variable | Overall CHILD Population | Manuscript | p-value (Manuscript vs. Overall) | Metagenomic | p-value (Metagenomic vs. Clinical) | Metabolomics | p-value (Metabolomics vs. Metagenomic) |
|---|---|---|---|---|---|---|---|
| No. patients | 3264 | 1115 | | 589 | | 509 | |
| Male, n(%) | | | 0.68 | | 0.57 | | 0.9 |
| | 1717 (52.6%) | 595 (53.4%) | | 323 (54.8%) | | 281 (55.2%) | |
| Ethnicity of Child, n(%) | | | 0.35 | | 1 | | 0.85 |
| Caucasian White | 2043 (63.6%) | 689 (62.1%) | | 365 (62.1%) | | 312 (61.4%) | |
| Non-Caucasian | 1167 (36.4%) | 421 (37.9%) | | 223 (37.9%) | | 196 (38.6%) | |
| Delivery Mode, n(%) | | | 0.79 | | 0.57 | | 0.99 |
| Vaginal | 2412 (74.8%) | 814 (74%) | | 421 (71.7%) | | 363 (71.6%) | |
| C-Section with labor | 425 (13.2%) | 146 (13.3%) | | 87 (14.8%) | | 74 (14.6%) | |
| C-Section without labor | 387 (12%) | 140 (12.7%) | | 79 (13.5%) | | 70 (13.8%) | |
| Breastfeeding status at 6 months, n(%) | | | 0.0091 | | 0.95 | | 1 |
| | 2323 (76.4%) | 884 (80.2%) | | 473 (80.4%) | | 408 (80.3%) | |
| Season of birth, n(%) | | | 0.85 | | 0.79 | | 0.9 |
| Spring | 889 (27.2%) | 293 (26.3%) | | 165 (28%) | | 146 (28.7%) | |
| Summer | 830 (25.4%) | 297 (26.6%) | | 145 (24.6%) | | 130 (25.5%) | |
| Fall | 755 (23.1%) | 259 (23.2%) | | 137 (23.3%) | | 120 (23.6%) | |
| Winter | 790 (24.2%) | 266 (23.9%) | | 142 (24.1%) | | 113 (22.2%) | |
| Atopy of father, n(%) | | | 0.84 | | 1 | | 0.94 |
| | 1663 (67.7%) | 622 (67.2%) | | 330 (67.3%) | | 289 (67.7%) | |
| Atopy of mother, n(%) | | | 0.068 | | 0.29 | | 1 |
| | 1727 (57.7%) | 668 (60.9%) | | 371 (63.5%) | | 321 (63.6%) | |
| Having older sibling, n(%) | | | 0.89 | | 0.3 | | 0.86 |
| | 1452 (45.9%) | 500 (45.6%) | | 282 (48.3%) | | 247 (49%) | |
| Antibiotics use in the first year of life, n(%) | | | 0.11 | | 0.8 | | 0.65 |
| | 605 (18.5%) | 231 (20.7%) | | 125 (21.2%) | | 102 (20%) | |
| NO2 in the first year of life | | | 0.23 | | 0.33 | | 0.87 |
| Median (Range) | 9.1 (0.5, 30.5) | 8.8 (1.2, 29.1) | | 9.1 (1.2, 29.1) | | 9 (1.2, 29.1) | |
| IQR (Q1,Q3) | 4.6, 13.3 | 4.5, 12.9 | | 4.7, 13.3 | | 4.6, 13.2 | |
| Birth weight Z-score | | | 0.76 | | 0.47 | | 0.74 |
| Median (Range) | -0.1 (-3.1, 4.3) | -0.1 (-3.1, 3.7) | | -0.1 (-2.6, 3.7) | | -0.1 (-2.6, 3.7) | |
| IQR (Q1,Q3) | -0.7, 0.5 | -0.7, 0.6 | | -0.7, 0.6 | | -0.7, 0.7 | |

**Supplementary Table. 1.** Demographic table between CHILD and sub-cohorts. For continuous variables, Wilcoxon tests were used for two groups, Kruskal-Wallis for more than two groups; fisher-exact tests were used for categorical variables, comparing the differences between the overall CHILD population, participants included within the manuscript, the subset of participants with metagenomic data, and participants with metabolomics data. The model comparing the larger CHILD cohort, and subsets of participants with metagenomic and metabolomic data included sex, ethnicity, delivery mode, breastfeeding status, season of birth, family history of atopy, family size, antibiotic usage in the first year of life, nitrogen oxide exposure, and birthweight.

| Variable | Adjusted odds ratio | Adjusted odds ratio confidence interval | P-value |
|---|---|---|---|
| Sex (male) | 1.84 | (1.36, 2.49) | 6.8E-05 |
| Ethnicity (Non-Caucasian) | 2.26 | (1.64, 3.1) | 5.1E-07 |
| C-section (with labor) | 0.71 | (0.45, 1.12) | 0.14 |
| C-section (without labor) | 1.56 | (0.96, 2.53) | 0.075 |
| Breastfeeding status at 6 months | 0.66 | (0.45, 0.99) | 0.043 |
| Season of birth (Summer) | 0.89 | (0.59, 1.34) | 0.59 |
| Season of birth (Fall) | 1.28 | (0.83, 1.97) | 0.27 |
| Season of birth (Winter) | 0.99 | (0.65, 1.5) | 0.96 |
| Paternal atopy | 1.56 | (1.13, 2.15) | 0.007 |
| Maternal atopy | 1.56 | (1.14, 2.12) | 0.0054 |
| Older sibling | 0.95 | (0.7, 1.3) | 0.75 |
| Antibiotics by 1 year | 2.25 | (1.55, 3.27) | 2E-05 |
| Birthweight Z-score | 1 | (0.85, 1.17) | 0.98 |
| Nitrogen dioxide IQR | 1.08 | (0.66, 1.77) | 0.76 |

**Supplementary Table 2.** Clinical and environmental factors linked to the development of allergic diagnoses at 5 years. Multivariable conditional logistic regression, using the data collection site as stratum, evaluating the odds ratio of developing one or more allergic disease by 5 years. The model comparing the groups of participants with and without allergic disease included sex, ethnicity, delivery mode, breastfeeding status, season of birth, family history of atopy, family size, antibiotic usage in the first year of life, nitrogen oxide exposure, and birthweight.

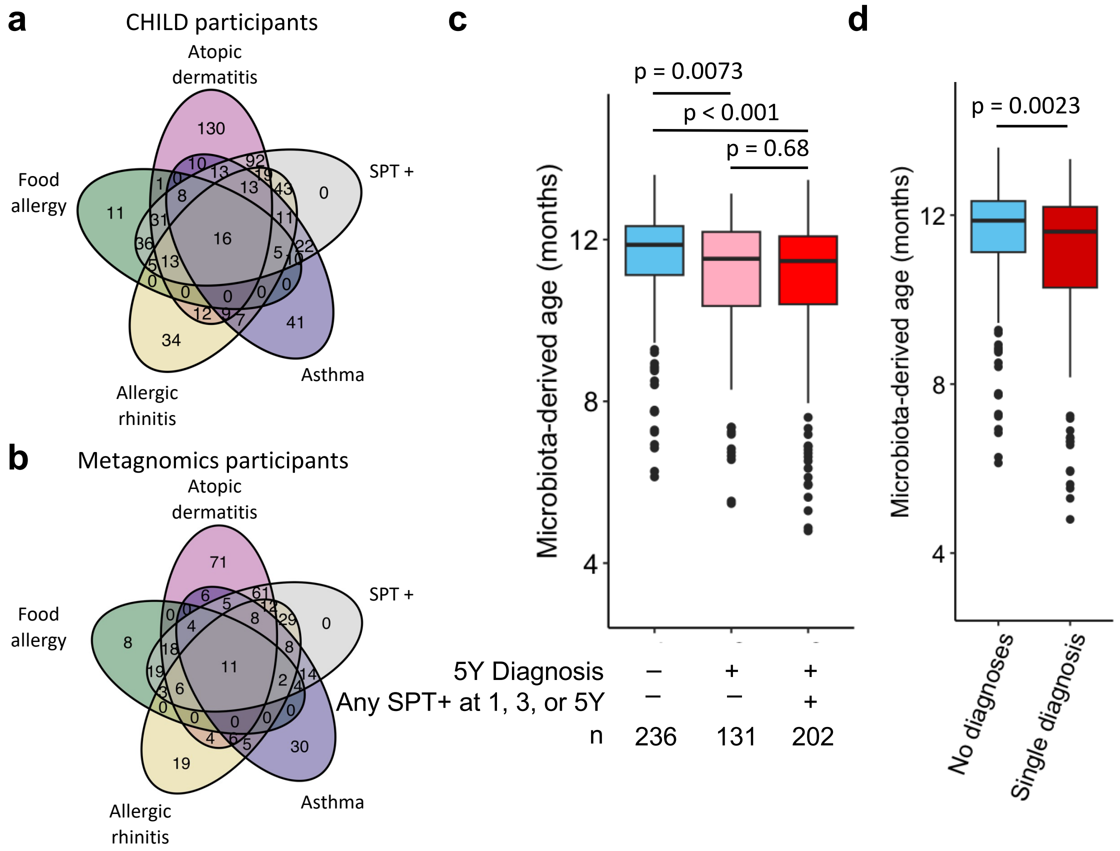


**Supplementary Fig. 1.** Atopy and allergic disease overlap amongst participants. Venn diagram of the overlap between participant diagnoses with atopic dermatitis (AD), food allergy (FA), allergic rhinitis (AR), asthma (As), and atopy (At) **a** in the CHILD cohort, **b** with shotgun metagenomics. Wilcoxon tests between the microbiota predicted ages of their 1-year microbiome samples of **c** non-allergic participants (n = 236) with participants diagnosed with atopy at any visit (1-, 3-, or 5-year evaluation) (n = 202, p = 0.0009), and those without atopy at any visit (n = 131), and **d** participants without diagnoses and those with only a single diagnosis (n = 353). For box plots, data are presented as box plots (centre line at the median, upper bound at 75th percentile, lower bound at 25th percentile) with whiskers at minimum and maximum values.

| Variables | Non-allergic | One or more allergic diagnoses | Adjusted Odds Ratio | P Value |
|---|---|---|---|---|
| No. participants | 231 | 348 | | |
| Male | | | | |
| | 105 (45.5%) | 211 (60.6%) | 1.9 (1.21, 2.96) | 0.0051 |
| Mode of delivery | | | | |
| Vaginal | 168 (73%) | 247 (71.2%) | Ref | --- |
| C-section with labor | 34 (14.8%) | 49 (14.1%) | 0.46 (0.24, 0.88) | 0.019 |
| C-section without labor | 28 (12.2%) | 51 (14.7%) | 1.04 (0.53, 2.04) | 0.9 |
| Paternal atopy | | | | |
| | 110 (58.5%) | 212 (72.6%) | 1.51 (0.95, 2.4) | 0.08 |
| Maternal atopy | | | | |
| | 124 (53.7%) | 242 (70.6%) | 1.67 (1.06, 2.62) | 0.027 |
| Breatfeeding status at 6 months | | | | |
| | 184 (79.7%) | 279 (80.4%) | 0.72 (0.4, 1.29) | 0.27 |
| Having an older sibling | | | | |
| | 129 (56.6%) | 149 (43.1%) | 0.68 (0.43, 1.08) | 0.1 |
| Antibiotic usage by 1 year | | | | |
| | 32 (13.9%) | 89 (25.6%) | 1.94 (1.11, 3.4) | 0.02 |
| Season | | | | |
| Spring | 66 (28.6%) | 97 (27.9%) | Ref | --- |
| Summer | 65 (28.1%) | 80 (23%) | 1.01 (0.57, 1.8) | 0.97 |
| Fall | 42 (18.2%) | 91 (26.1%) | 1.56 (0.82, 2.95) | 0.17 |
| Winter | 58 (25.1%) | 80 (23%) | 1.08 (0.59, 1.99) | 0.79 |
| Birth weight (Z-score) | | | | |
| Median (Range) | 0 (-2.6, 3.2) | -0.2 (-2.5, 3.7) | 0.95 (0.75, 1.2) | 0.65 |
| Ethnicity | | | | |
| Caucasian White | 175 (75.8%) | 182 (52.4%) | Ref | --- |
| Non-Caucasian | 56 (24.2%) | 165 (47.6%) | 2.23 (1.36, 3.65) | 0.0015 |
| IQR increase in Nitrogen Dioxide | | | | |
| Median (Range) | 0.8 (0.2, 2.5) | 1.2 (0.1, 3.4) | 1.04 (0.49, 2.21) | 0.92 |
| IQR increase in predicted age | | | | |
| Median (Range) | 7.8 (4, 9) | 7.6 (3.2, 8.9) | 0.75 (0.59, 0.94) | 0.013 |

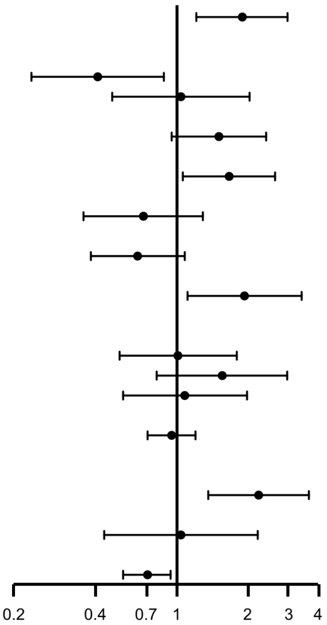


**Supplementary Fig. 2.** Predicted age remains protective when accounting for covariates. Multivariable conditional logistic regression, using the data collection site as stratum, evaluating the odds ratio of developing one or more atopic or allergic diagnoses (n = 348) when accounting for early-life and familial exposures as compared to non-allergic children (n = 231). Data are presented as adjusted odds ratios (95% confidence intervals).

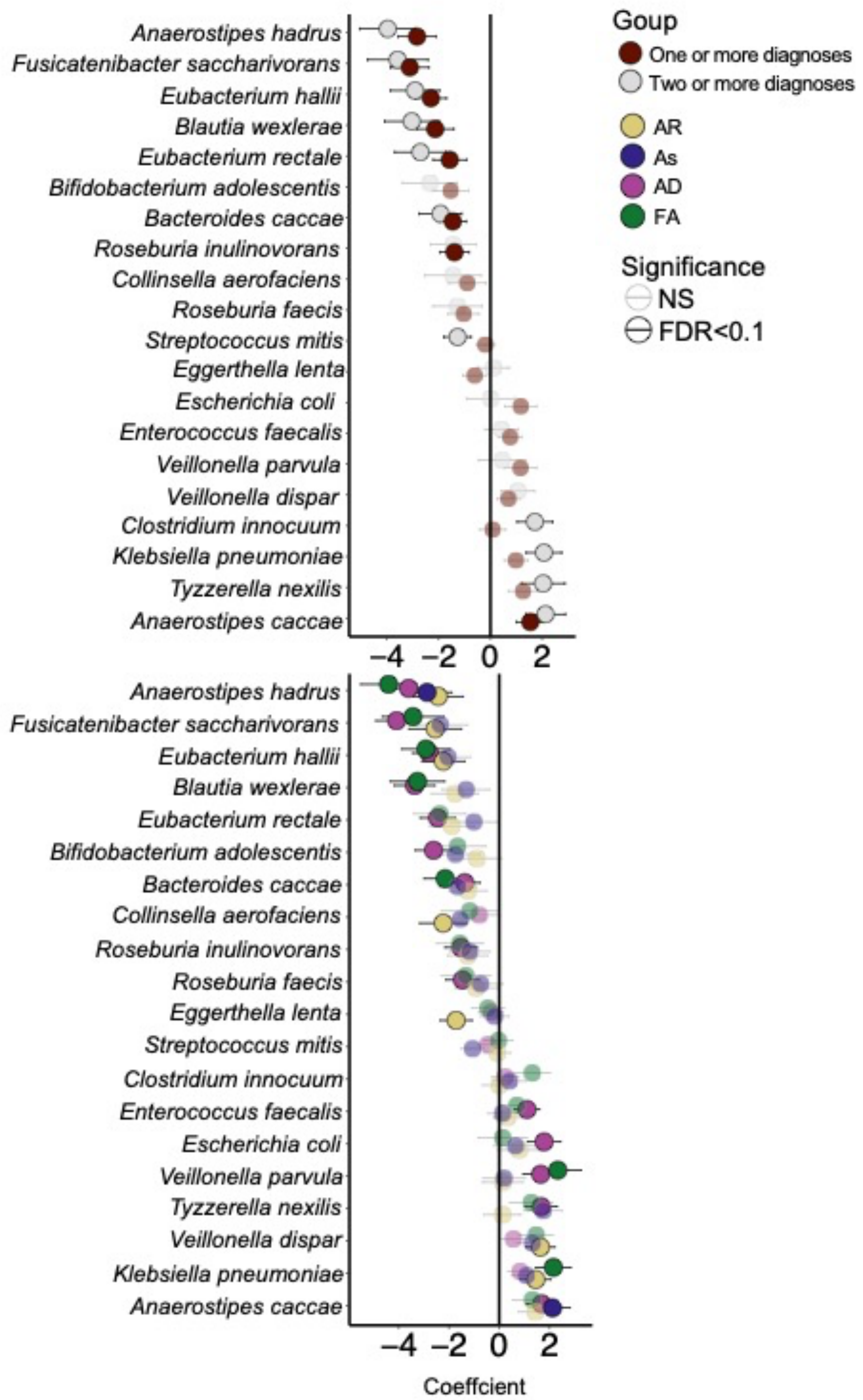


**Supplementary Fig. 3.** Important underlying infant microbiota of allergic disease. The 20 commonly identified species within one or more atopic or allergic diagnoses (n = 353), two or more allergic diagnoses (n = 82), and individual clinical diagnoses at 5 years, i.e., atopic dermatitis (AD, n = 212), food allergy (FA, n = 75), asthma (As, n = 103), or allergic rhinitis (AR, n = 113), at 5 years, and healthy control (n = 236) participants, adjusting for chronological age at the time of collection and with a random effect of the sample collection site. Data are presented as MaAslin2 coefficients +/- standard error.

| Species | Variable | Coefficient | St. err. | P-Value | FDR |
|---|---|---|---|---|---|
| *Megasphaera micronuciformis* | Breastfeeding at 6 months | 0.71 | 0.10 | 0.00 | 0.00 |
| *Veillonella atypica* | Breastfeeding at 6 months | 0.64 | 0.11 | 0.00 | 0.00 |
| *Tyzzerella nexilis* | Breastfeeding at 6 months | -0.55 | 0.11 | 0.00 | 0.00 |
| *Clostridium innocuum* | Breastfeeding at 6 months | -0.45 | 0.10 | 0.00 | 0.00 |
| *Intestinibacter bartlettii* | Breastfeeding at 6 months | -0.37 | 0.10 | 0.00 | 0.00 |
| *Ruminococcus gnavus* | Breastfeeding at 6 months | -0.33 | 0.09 | 0.00 | 0.00 |
| *Sellimonas intestinalis* | Breastfeeding at 6 months | -0.41 | 0.12 | 0.00 | 0.00 |
| *Erysipelatoclostridium ramosum* | Breastfeeding at 6 months | -0.29 | 0.11 | 0.01 | 0.01 |
| *Eggerthella lenta* | Breastfeeding at 6 months | -0.20 | 0.07 | 0.01 | 0.02 |
| *Enterococcus faecalis* | Breastfeeding at 6 months | -0.22 | 0.09 | 0.02 | 0.03 |
| *Escherichia coli* | Breastfeeding at 6 months | 0.29 | 0.12 | 0.02 | 0.03 |
| *Gordonibacter pamelaeae* | Breastfeeding at 6 months | -0.19 | 0.09 | 0.04 | 0.07 |
| *Ruminococcus bromii* | Breastfeeding at 6 months | -0.25 | 0.12 | 0.04 | 0.07 |
| *Clostridium innocuum* | Paternal atopy | -0.60 | 0.24 | 0.01 | 0.05 |
| *Erysipelatoclostridium ramosum* | Paternal atopy | -0.56 | 0.25 | 0.02 | 0.07 |
| *Bifidobacterium longum* | Antibiotic usage by 1 year | -1.54 | 0.40 | 0.00 | 0.00 |
| *Tyzzerella nexilis* | Antibiotic usage by 1 year | 1.01 | 0.27 | 0.00 | 0.00 |
| *Clostridium innocuum* | Antibiotic usage by 1 year | 0.73 | 0.25 | 0.00 | 0.01 |
| *Veillonella atypica* | Antibiotic usage by 1 year | -0.80 | 0.27 | 0.00 | 0.01 |
| *Sellimonas intestinalis* | Antibiotic usage by 1 year | 0.81 | 0.29 | 0.01 | 0.02 |
| *Hungatella hathewayi* | Antibiotic usage by 1 year | 0.78 | 0.30 | 0.01 | 0.02 |
| *Megasphaera micronuciformis* | Antibiotic usage by 1 year | -0.53 | 0.24 | 0.03 | 0.06 |

**Supplementary Table 3.** Microbiota associated with important clinical features in allergic disease. MaAsLin2 model, adjusting for chronological age at the time of collection and with a random effect of the sample collection site, results indicating the microbe identified as significant, the variable and comparison group, coefficient of association, standard deviation, p-value, and FDR-corrected p-value.

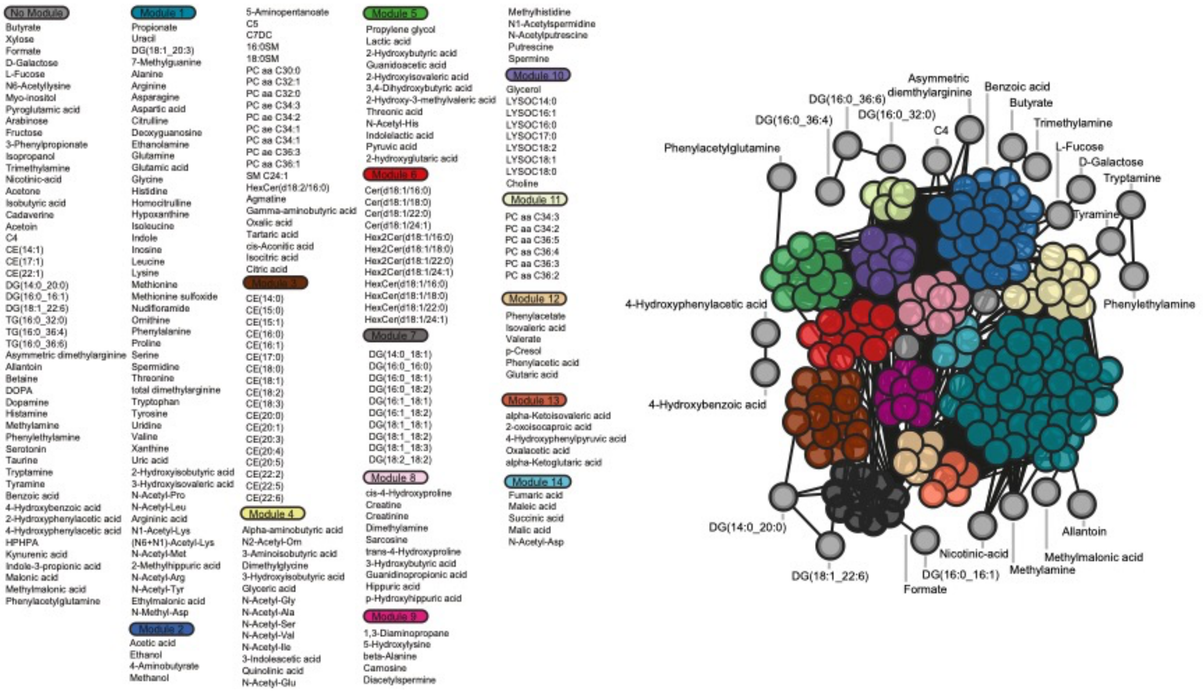


**Supplementary Fig. 4.** Metabolic components in clusters identified via weighted correlation analysis. Weighted gene co-expression analysis (WGCNA) was used to cluster 195 quantified metabolites through targeted liquid chromatography with tandem mass spectrometry and nuclear magnetic resonance into 14 clusters, with 50 metabolites not assigned a cluster. The correlations between individual cluster metabolites and independent clusters with no modules are depicted by a network plot generated using Cytoscape and metabolite cluster components are listed along the sides of the figure.

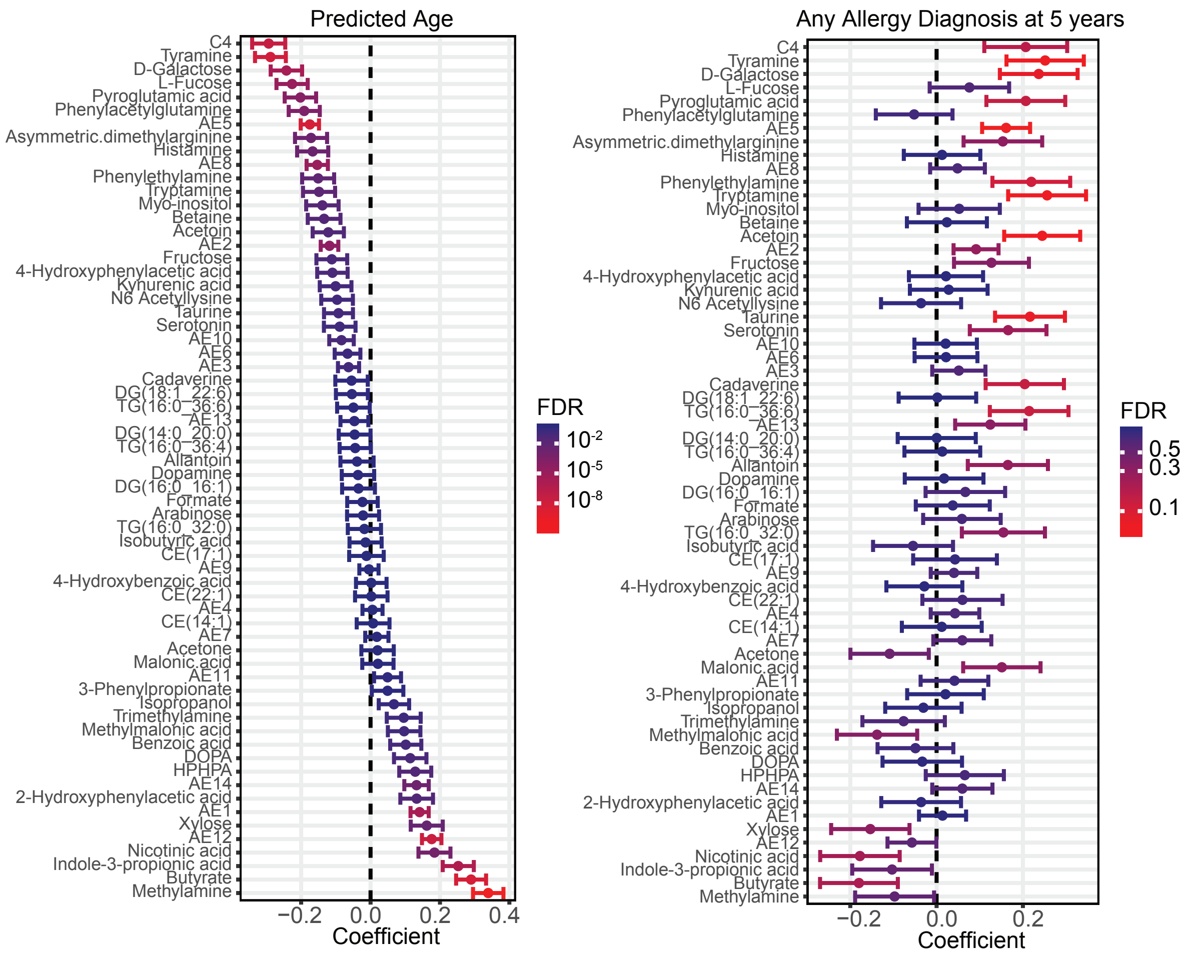


**Supplementary Fig. 5.** Metabolites linked to predicted age and allergic disease. MaAsLin2 results of metabolites associated with a predicted age (n = 509) and b one or more allergic disease (n = 305) as compared to participants with no allergic history (n = 204). Data are presented as MaAslin2 coefficients +/- standard error of the mean.
