# Supplementary material for: Delayed gut microbiota maturation in the first year of life is a hallmark of pediatric allergic disease
Source: Nat Commun. 2023 Aug 29;14:4785. doi: 10.1038/s41467-023-40336-4 (PMC10465508; doi:10.1038/s41467-023-40336-4)
Supplement: Supplementary file 3 — Description of Additional Supplementary Files [file 41467_2023_40336_MOESM3_ESM.docx]

**Hoskinson et al., “Delayed gut microbiota maturation in the first year of life is a hallmark of pediatric allergic disease”**

**Description of Additional Supplementary Files**

File Name: Supplementary Data 1

Description: Clinical and environmental factors linked to the development of allergic diagnoses at 5 years. Multivariable conditional logistic regression, using the data collection site as a stratum, evaluates the odds ratio of developing one or more allergic diseases, two or more allergic diseases, atopic dermatitis, food allergy, allergic rhinitis, and/or asthma by 5 years. The model comparing the groups of participants with and without allergic disease included sex, ethnicity, delivery mode, breastfeeding status, season of birth, family history of atopy, family size, antibiotic usage in the first year of life, nitrogen oxide exposure, and

birthweight.

File Name: Supplementary Data 2

Description: Metacyc pathways associated with allergic diagnoses and allergic diseases. MaAsLin2 model, adjusting for chronological age at the time of collection and with a random effect of the sample collection site, results indicating the pathway identified as significant, the variable and comparison group, coefficient of association, standard deviation, p-value, and FDR-corrected p-value.

File Name: Supplementary Data 3

Description: 11 Metacyc pathways associated with both allergic diagnoses and allergic diseases. MaAsLin2 model, adjusting for chronological age at the time of collection and with a random effect of the sample collection site, results indicating the pathway identified as significant, the variable and comparison group, coefficient of association, standard deviation, p-value, and FDR-corrected p-value.

File Name: Supplementary Data 4

Description: MaAsLin2 results of all metabolite associations with an allergy outcome. Analysis of whether metabolites removed through filtering are missing at random.
