# Supplementary material for: Delayed gut microbiota maturation in the first year of life is a hallmark of pediatric allergic disease
Source: Nat Commun. 2023 Aug 29;14:4785. doi: 10.1038/s41467-023-40336-4 (PMC10465508; doi:10.1038/s41467-023-40336-4)
Supplement: Supplementary file 8 — Reporting Summary [file 41467_2023_40336_MOESM8_ESM.pdf]

nature portfolio

Corresponding author(s): Stuart E. Turvey (sturvey@bcchr.ca)

Last updated by author(s): Jul 14, 2023

Reporting Summary

Nature Portfolio wishes to improve the reproducibility of the work that we publish. This form provides structure for consistency and transparency in reporting. For further information on Nature Portfolio policies, see our Editorial Policies and the Editorial Policy Checklist.

Statistics

For all statistical analyses, confirm that the following items are present in the figure legend, table legend, main text, or Methods section.

n/a	Confirmed	
☐	☒	The exact sample size (n) for each experimental group/condition, given as a discrete number and unit of measurement
☐	☒	A statement on whether measurements were taken from distinct samples or whether the same sample was measured repeatedly
☐	☒	The statistical test(s) used AND whether they are one- or two-sided<br>*Only common tests should be described solely by name; describe more complex techniques in the Methods section.*
☐	☒	A description of all covariates tested
☐	☒	A description of any assumptions or corrections, such as tests of normality and adjustment for multiple comparisons
☐	☒	A full description of the statistical parameters including central tendency (e.g. means) or other basic estimates (e.g. regression coefficient) AND variation (e.g. standard deviation) or associated estimates of uncertainty (e.g. confidence intervals)
☐	☒	For null hypothesis testing, the test statistic (e.g. F, t, r) with confidence intervals, effect sizes, degrees of freedom and P value noted<br>*Give P values as exact values whenever suitable.*
☒	☐	For Bayesian analysis, information on the choice of priors and Markov chain Monte Carlo settings
☒	☐	For hierarchical and complex designs, identification of the appropriate level for tests and full reporting of outcomes
☐	☒	Estimates of effect sizes (e.g. Cohen's d, Pearson's r), indicating how they were calculated

Our web collection on statistics for biologists contains articles on many of the points above.

Software and code

Policy information about availability of computer code

Data collection	CHILD utilizes REDCap to record clinical data. REDCap is a secure web application for building and managing online surveys and databases. While REDCap can be used to collect virtually any type of data in any environment (including compliance with 21 CFR Part 11, FISMA, HIPAA, and GDPR), it is specifically geared to support online and offline data capture for research studies and operations. The REDCap Consortium, a vast support network of collaborators, is composed of thousands of active institutional partners in over one hundred countries who utilize and support their own individual REDCap systems.
Data analysis	Data analysis was conducted in R (version 4.1.1). The bioBakery 3 pipeline was used to map sequences and classify sequences into taxonomic (species and strain level) and functional features within each sample 46. The bioBakery 3 pipeline is open source and its functionality has been explained in published detail. Specifically, MetaPhlAn 3 was used for taxonomic classification, and HUMAnN 3 for functional profiling. "RandomForest", "mlbench", and "caret" packages were used to generate predicted age. The "phyloseq" package was used to pre-process the metagenomic taxonomy table. The "Maaslin2" package was used to perform linear mixed-effects models (MaAsLin2 function) with study center location as a random effect and adjusting for stool sample of collection age. Spearman correlation analyses were performed using the "RcmdrMisc" package and reported using r and Benjamini-Hochberg-corrected p-values. Permutational Multivariate Analysis of Variance (PERMANOVA) analysis was applied to quantify the association between infant microbiota-derived predicted age and metabolome using the R package "vegan". To evaluate the mediation effect of dysregulated pathways and metabolites for gut maturity on atopic diseases, we applied structural equation modeling (SEM) using the R package "lavaan". We have attached additional files of each script for each figure, including the code used to generate the predicted age in Fig. 2.<br><br>Version: 3.10.0 Java: 17.0.5 by Eclipse Adoptium<br>Java Home: /Applications/Cytoscape_v3.10.0/.install4j/jre.bundle/Contents/Home<br>OS: Mac OS X 11.5.2 - x86_64

Detailed R environment and package versions utilized:
R version 4.1.1 (2021-08-10)
Platform: x86_64-apple-darwin17.0 (64-bit)
Running under: macOS Big Sur 11.5.2

Matrix products: default
LAPACK: /Library/Frameworks/R.framework/Versions/4.1/Resources/lib/libRlapack.dylib

locale:
[1] en_US.UTF-8/en_US.UTF-8/en_US.UTF-8/C/en_US.UTF-8/en_US.UTF-8

attached base packages:
[1] grid stats4 stats graphics grDevices utils datasets methods base

other attached packages:
dplyr_1.1.0
forestplot_3.1.1
vegan_2.6-4
reshape2_1.4.4
corrplot_0.92
ggpubr_0.6.0
lavaan_0.6-15
Maaslin2_1.8.0
caret_6.0-93
ggplot2_3.4.1
mlbench_2.1-3
randomForest_4.7-1.1
RColorBrewer_1.1-3

For manuscripts utilizing custom algorithms or software that are central to the research but not yet described in published literature, software must be made available to editors and reviewers. We strongly encourage code deposition in a community repository (e.g. GitHub). See the Nature Portfolio guidelines for submitting code & software for further information.

Data

Policy information about availability of data

All manuscripts must include a data availability statement. This statement should provide the following information, where applicable:

- Accession codes, unique identifiers, or web links for publicly available datasets
- A description of any restrictions on data availability
- For clinical datasets or third party data, please ensure that the statement adheres to our policy

The accession numbers for the shotgun metagenomic data reported in this paper are BioProject accession (NCBI): PRJNA838575.

The nuclear magnetic resonance (NMR) and liquid chromatography with tandem mass spectrometry (LC-MS/MS) data is deposited in MetaboLights with the accession number MTBLS7919.

Code for main figures is provided and other code is available upon request.

For infant stool data, further requests for resources and reagents should be directed to and will be fulfilled by Stuart E. Turvey (sturvey@bcchr.ca).

Human research participants

Policy information about studies involving human research participants and Sex and Gender in Research.

Reporting on sex and gender

Our findings are equally applicable to both male and female biological sexes. Although gender will most likely play a role in CHILD studies as the participants age and mature, no self- or parental-reporting of gender was used in this study and none was collected and is unavailable to report. Participants were of infant age and were thus not yet able to report any gener identification that differed from their biological sex. Within the current study, biological sex was used as a covariate based upon previous reports in the literature demonstrating differences in allergic incidence between biologically male or female individuals. Parents of participants provided consent for the anonymous reporting of clinical and biological data of participants and the proportion of biologically male and female participants has been reported in the initial multivariate conditional regression tables.

Population characteristics

We derived variables indicating whether participants were diagnosed with a condition of interest or had no conditions up and through their 5-year evaluation and used multivariable conditional logistic regression (stratified by study center) to evaluate the influence of early-life and familial exposures, including biological sex, presence of older siblings, mode of delivery at birth, birth weight, the season of birth, breastfeeding status at the age of 6 months, maternal atopy, paternal atopy, and exposure to environmental NO2, upon atopic condition development by the age of 5 years. Missing data were considered missing

completely at random and individuals were removed from the multivariable analysis if they had a missing value in any covariates.

Overall CHILD population:
n = 3,264
Age: Mean 1.05 years SD 0.15
Biological sex: Male n = 1,717, Female n = 1,547
Ethnicity: Caucasian n = 2,043, Non-Caucasian n = 1,167
Delivery mode: Vaginal n = 2,412, C-section with labor n = 425, C-section without labor n = 387
Breastfeeding at 6 months: Yes n = 2,323, No n = 941
Season of birth: Spring n = 889, Summer n = 830, Fall n = 755, Winter n = 790
Atopic father: Yes n = 1,663, No n = 1,601
Atopic mother: Yes n = 1,727, No n = 1,537
Older sibling: Yes n = 1,452, No n = 1,812
Antibiotics: Yes n = 605, No n = 2,659
NO2 in the first year of life: Median 9.1 (0.5, 30.5)
Birth weight Z-score: Median -0.1 (-3.1, 4.3)

CHILD population subset included in manuscript:
n = 1,115
Age: Mean 1.04 years SD 0.13
Biological sex: Male n = 595, Female n = 520
Ethnicity: Caucasian n = 689 Non-Caucasian n = 426
Delivery mode: Vaginal n = 814, C-section with labor n = 146, C-section without labor n = 140
Breastfeeding at 6 months: Yes n = 884, No n = 231
Season of birth: Spring n = 293, Summer n = 297, Fall n = 259, Winter n = 266
Atopic father: Yes n = 622, No n = 493
Atopic mother: Yes n = 668, No n = 447
Older sibling: Yes n = 500, No n = 615
Antibiotics: Yes n = 231 No n = 884
NO2 in the first year of life: Median 8.8 (1.2, 29.1)
Birth weight Z-score: Median -0.1 (-3.1, 3.7)

Recruitment

With enrolment beginning in 2008 and closing in 2012, a total of 3621 pregnant women from four cities (Vancouver, Edmonton, Winnipeg, Toronto) across Canada enrolled along with eligible infants (n=3455) that had no congenital abnormalities and were born at a minimum of 34 weeks of gestation.

CHILD Study children were followed prospectively and detailed information on environmental exposures and clinical measurements and assessments were collected using a combination of questionnaires and in-person clinical assessments. Briefly, questionnaires were administered at recruitment, 36-week gestation, at 3, 6, 12, 18, 24, 30 months, and at 3, 4, and 5 years; data were obtained related to environmental exposures and general health. In addition, at ages 1, 3, and 5 years, questionnaires validated in the International Study of Asthma and Allergies in Childhood (ISAAC) were completed by the parent.

Self-selection bias potentially occurred at the socioeconomic level. Families and children with more time due to fewer working hours or expendable income, as well as those with more resources and higher education to be aware of the CHILD study may potentially skew study results toward a higher income demographic.

Ethics oversight

University of British Columbia, University of Manitoba, University of Toronto, McMaster University, BC Children's Hospital, The Hospital for Sick Children, Simon Fraser University.

REB Number: H07-03120

This ethics approval applies to research ethics issues only and does not include provision for any administrative approvals required from individual institutions before research activities can commence.
The Board of Record (as noted above) has reviewed and approved this study in accordance with the requirements of the Tri-Council Policy Statement: Ethical Conduct for Research Involving Humans (TCPS2, 2018).
The "Board of Record" is the Research Ethics Board delegated by the participating REBs involved in a harmonized study to facilitate the ethics review and approval process.

This study has been approved either by the Board of Record’s full REB or by an authorized delegated reviewer.

Note that full information on the approval of the study protocol must also be provided in the manuscript.

Field-specific reporting

Please select the one below that is the best fit for your research. If you are not sure, read the appropriate sections before making your selection.

☒ Life sciences ☐ Behavioural & social sciences ☐ Ecological, evolutionary & environmental sciences

For a reference copy of the document with all sections, see nature.com/documents/nr-reporting-summary-flat.pdf

Life sciences study design

All studies must disclose on these points even when the disclosure is negative.

Sample size

The CHILD study is a representative population-based cohort that recruited as many mothers as was feasible. CHILD is a longitudinal, general population birth cohort study following infants from mid-pregnancy to age 5 years. Over this time period, biological samples, questionnaires, clinical measures and environmental data are collected (Moraes et al., 2015. Paediatric and Perinatal Epidemiology).

No predetermined sample sizes were selected overall, but samples sent in for shotgun metagenomic sequencing were selected at a proportion so that the % of participants who had developed asthma was reflective of the Canadian population (5-7%).

Within the current study, sample size was primarily determined by the size of CHILD and our exclusion of participants based upon missing data or transient atopic tendencies. The CHILD study is an observational study and thus depends on available data.

The primary outcomes of our study were atopic dermatitis, asthma, food allergy, and allergic rhinitis diagnosed (as Yes/Possible/No), using physical symptoms in combination with skin prick testing, by an expert study physician at the clinical assessment at the age of 5 years based on our published approach. For this study, children were considered to have atopic or allergic diseases only if the response was 'Yes' and the phenotype was defined as comparing children with 'Yes' responses at 5 years.

Non-allergic controls were limited to children with 'No' responses for 5-year diagnoses, negative allergen SPTs at 1, 3, and 5 years, and no history of wheezing at 1, 3, and 5 years. Within the current study, we analyzed the data in a subset of CHILD that contained data for parent and child questionnaires, SPT results, and physician diagnoses at 5 years for cases and 1, 3, and 5 years for controls (n =1115, Supplementary Fig. 1). These outcomes are clinically defined and determined our respective samples sizes. Further, samples that did not meet quality control standards by the data-generating cores that produced the sequencing data were removed from our analysis. Our approach resulted in a suitable sample size based upon the population and observational nature of our study with supplemental data analysis that provides insight into general trends on a large scale.

Data exclusions

Data exclusions were pre-established: participants who were not diagnosed with our primary outcomes of interest but had positive (>2mm) skin prick tests to allergens at any of their 3 visits at 1, 3, or 5 years were removed from our analysis, participants who were not diagnosed with our primary outcomes of interest but had missing data at any of their 3 visits at 1, 3, or 5 years were removed, samples that did not reach quality standards of the sequencing and metabolomic cores were removed, samples with <1million sequencing reads were removed, and, regarding clinical and epidemiological analysis, missing data were considered missing completely at random and individuals were removed from the multivariable analysis if they had a missing value in any covariates.

Replication

Technical replicates were not included within this analysis. Shotgun metagnomic sequencing, nuclear magnetic resonance, or liquid chromatography with tandem mass spectrometry were not replicated for individual stool samples. Experiments were performed, one time each, on longitudinal samples from the same individual (2 stool samples collected at 3 months and 1 year, respectively).

Longitudinally-collected samples (i.e., 3-month and 1-year samples analyzed via shotgun metagenomic sequencing) were collected from the same children over time. However, there were no redundant data points from the same participants within our analysis of 1-year samples. in other words, samples collected at the one year time point were not repeatedly analyzed using shotgun metagenomic sequencing and each participant has only one corresponding sample data point.

In addition, there was also no redundant data points from the same participants within the metabolomics data. The metabolomics data was obtained from a subset of samples from the same participants who's stool samples were analyzed by shotgun metagenomic sequencing, thus providing a holistic analysis of the gut microbiomes of said participants with complementary biological analyses rather than replicated data points. Our analyses are entirely reproducible with our dataset, as can be seen with the available code provided.

However, we do note that there is overlap between children diagnosed with each respective disease (e.g., some children have both atopic dermatitis and food allergy but are included within each respective group.

Randomization

This is not an intervention study, but rather a prospective longitudinal observational study. Samples were allocated into their respective groups based solely on their clinical diagnoses by clinicians at their 1, 3, and 5 year evaluations. However, confounding variables such as study site were adjusted for in models to ensure that any skewing of imbalanced population statistics would be adjusted for.

Blinding

Blinding was not possible on the part of the physicians as they evaluated participants for clinical symptoms of allergy, but sequencing and metabolite quantification were performed without bias as to which sample belonged to non-allergic or allergic participants.

Reporting for specific materials, systems and methods

We require information from authors about some types of materials, experimental systems and methods used in many studies. Here, indicate whether each material, system or method listed is relevant to your study. If you are not sure if a list item applies to your research, read the appropriate section before selecting a response.

Materials & experimental systems

n/a	Involved in the study
☒	☐ Antibodies
☒	☐ Eukaryotic cell lines
☒	☐ Palaeontology and archaeology
☒	☐ Animals and other organisms
☐	☒ Clinical data
☒	☐ Dual use research of concern

Methods

n/a	Involved in the study
☒	☐ ChIP-seq
☒	☐ Flow cytometry
☒	☐ MRI-based neuroimaging

Clinical data

Policy information about clinical studies

All manuscripts should comply with the ICMJEguidelines for publication of clinical research and a completedCONSORT checklist must be included with all submissions.

Clinical trial registration

N/A; Population cohort study; Approval for current study obtained through the University of British Columbia, Vancouver, Canada, Certification #: H07-03129 , B17-0193

Study protocol

A detailed published protocol of the CHILD cohort by Moraes et al. can be found in the journal Paediatric and Perinatal Epidemiology (https://doi.org/10.1111/ppe.12161).

Data collection

CHILD Study children were followed prospectively and detailed information on environmental exposures and clinical measurements and assessments were collected using a combination of questionnaires and in-person clinical assessments. Briefly, questionnaires were administered at recruitment, 36-week gestation, at 3, 6, 12, 18, 24, 30 months, and at 3, 4, and 5 years; data were obtained related to environmental exposures and general health. In addition, at ages 1, 3, and 5 years, questionnaires validated in the International Study of Asthma and Allergies in Childhood (ISAAC) were completed by the parent. All infants enrolled in the CHILD protocol were administered a skin prick test at their 1-, 3-, and 5-year scheduled visits. Children were then diagnosed with IgE-mediated allergic sensitization (also referred to as atopy) based on skin prick testing (SPT) to multiple common food and environmental inhalant allergens, using ≥2 mm average wheal size as indicating a positive test relative to the negative control. Allergens tested at the 1-, 3-, and 5-year visit were German cockroach, Alternaria alternata, house dust mites (Dermatophagoides psteronyssinus and Dermatophagoides farnae), cat hair, dog epithelium, cow's milk, peanut, egg white, and soybean. Glycerin and histamine served as the negative and positive controls, respectively.

Sample collection and sequencing were performed as previously described. Specifically, stool samples from diapers were collected at a home visit at around 3 months [mean (SD), 3.8 (1.1) months] and a clinic visit at around 1 year [mean (SD), 12.5 (1.6) months]. Samples were aliquoted into four 2-mL cryovials using a stainless steel depyrogenated spatula and were frozen at −80°C. Shotgun metagenomic sequencing data were generated by Diversigen (Minneapolis, MN, USA) from fecal samples (average depth of 5 million reads per sample). DNA was extracted from samples using the MO BIO PowerSoil Pro with bead beating in 0.1mm glass bead plates, with high-quality input DNA verified using Quant-iT PicoGreen. Libraries were prepared and sequenced on an Illumina NextSeq using single-end 1 x 150 reads. Low-quality (Q-Score<30) and length (<50) sequences were removed, and adapter sequences were trimmed. Host and low-quality reads were removed, and only samples with at least 1 million remaining reads or more were retained for downstream analysis.

A subset of the same stool samples that were sequenced was then analyzed at The Metabolomics Innovation Centre in Edmonton, Alberta for metabolites by targeted nuclear magnetic resonance (NMR) and liquid chromatography with tandem mass spectrometry (LC-MS/MS). Briefly, each analysis was performed using approximately 100mg of stool. Samples with low mass or diaper fibers were excluded. Stool was weighed before and after lyophilization to quantify total water content prior to analysis. Analyte concentrations were determined using a standard approach to absolute quantification, using isotope-labeled internal standards to correct for technical variation and then assessing the result against a calibration curve of known concentrations of standard mixtures. Regarding the assignment of all visible peaks, this applies to when, within this targeted assay, very low abundant peaks are not visible for manual assignment and are therefore not reported Metabolite levels (µmol) were normalized to dry/lyophilizeed stool weight (g) and analyzed using the ratio (µmol/g). All metabolite concentrations for both the NMR and LC-MS/MS analysis were recorded by TMIC as well as their limit of detection (LOD).

Outcomes

CHILD was designed to collect data pertaining to allergy and general health. Therefore, pre-defined outcomes of the study as a whole included defining the early-life influences on allergy and asthma, such as allergen exposure, familial structure, birth weight, genetic susceptibility to atopy, etc. Within the evaluations of clinicians they performed general clinical assessments where they reported the existence of aptopic symptoms. Thus, we were able to use these assessments as he primary outcomes of our study. We used atopic dermatitis, asthma, food allergy, and allergic rhinitis diagnosed (as Yes/Possible/No), using physical symptoms in combination with skin prick testing, by an expert study physician at the clinical assessment at the age of 5 years based on our published approach 45. For this study, children were considered to have atopic or allergic diseases only if the response was 'Yes' and the phenotype was defined as comparing children with 'Yes' responses at 5 years. Non-allergic controls were limited to children with 'No' responses for

5-year diagnoses, negative allergen SPTs at 1, 3, and 5 years, and no history of wheezing at 1, 3, and 5 years. Within the current study, we analyzed the data in a subset of CHILD that contained data for parent and child questionnaires, SPT results, and physician diagnoses at 5 years for cases and 1, 3, and 5 years for controls (n =1115).
